# Evaluation of Inflammation Caused by Cardiopulmonary Bypass in a Small Animal Model

**DOI:** 10.3390/biology9040081

**Published:** 2020-04-20

**Authors:** Yutaka Fujii

**Affiliations:** Department of Clinical Engineering and Medical Technology, Niigata University of Health and Welfare, Niigata 950-3198, Japan; fujii@nuhw.ac.jp

**Keywords:** systemic inflammation, cytokine, oxidative stress extracorporeal circulation, cardiopulmonary bypass

## Abstract

Extracorporeal circulation (ECC) methods are being increasingly used for mechanical support of respiratory and cardio-circulatory failure. Especially, cardiopulmonary bypass (CPB) during cardiovascular surgery, sustenance of the patient’s life by providing an appropriate blood flow and oxygen supply to principal organs. On the other hand, systemic inflammatory responses in patients undergoing cardiovascular surgery supported by CPB contribute significantly to CPB-associated mortality and morbidity. Our previous research showed that CPB causes a systemic inflammatory response and organ damage in a small animal CPB model. We have been studying the effects of hyperoxia and blood plasma substitute on CPB. In this review, we present a study focusing on the systemic inflammatory response during CPB, along with our findings.

## 1. Introduction

Extracorporeal circulation (ECC) methods are being increasingly used for mechanical support of respiratory and cardio-circulatory failure. Especially, cardiopulmonary bypass (CPB) during cardiovascular surgery, sustenance of the patient’s life by providing an appropriate blood flow and oxygen supply to principal organs. [1]. Until the 1950s, cardiovascular surgery had a very high mortality and morbidity rate. This situation changed due to the development of the CPB system by Dr. Gibbon [2] and body surface cooling for hypothermia by Dr. Gordon [3]. These inventions established modern cardiovascular surgery.

However, it has been known that CPB initiates an inflammatory reaction cascade. Cardiovascular surgery with CPB is often associated with a systemic inflammatory response syndrome, significantly affecting the postoperative mortality and morbidity [1,4,5]. The inflammatory response caused by CPB is known to especially affect humans [6,7].

There are several factors that appear to be cause for the systemic inflammatory reaction, such as contact of blood with the CPB device’s surface, surgical operation trauma, endotoxemia, blood loss, and ischemic reperfusion injury [8]. Accordingly, activation of the complement and immune system, leucocytes, and endothelial cells occurs, which in turn is responsible for the release of multiple pro-inflammatory cytokines [9]. The increase in cytokines, as in interleukins (IL), tumor necrosis factor (TNF), kallikrein, and bradykinin [10], exacerbates the inflammatory response during cardiovascular surgery with CPB [11]. Inflammation during extracorporeal circulation causes systemic inflammatory response syndrome and induces serious complications [12,13]. Kidney injury occurs especially frequently, and it is closely related to inflammation during cardiopulmonary bypass [14,15,16]. In fact, looking at clinical data, acute kidney injury (AKI) occurs in 5% of admitted patients [17] and in 5–30% of patients undergoing cardiovascular surgery, who have a high mortality and morbidity rate [17,18]. 

Moreover, inflammation and arrhythmia after cardiac surgery are also highly related. Myocardial reperfusion after aortic cross unclamping generates enormous stress and might activate leukocytes. Patients who have higher postoperative leukocyte counts are significantly more likely to develop postoperative atrial fibrillation [19,20,21,22], and patients developing postoperative atrial fibrillation tend to have a greater degree of monocyte-macrophage lineage activation, as reflected by high expression of CD11b [23]. 

In addition, cardiac surgery with CPB results in generation of active oxygen species (oxidative stress) and coagulopathy. Eventually, these phenomena can cause failure of multiple organs and catastrophic complication.

CPB causes a variety of catastrophic complications, and leukocyte activation, especially neutrophils and monocytes, results in worse outcomes for patients undergoing cardiac surgery with CPB. 

**Figure 1:**
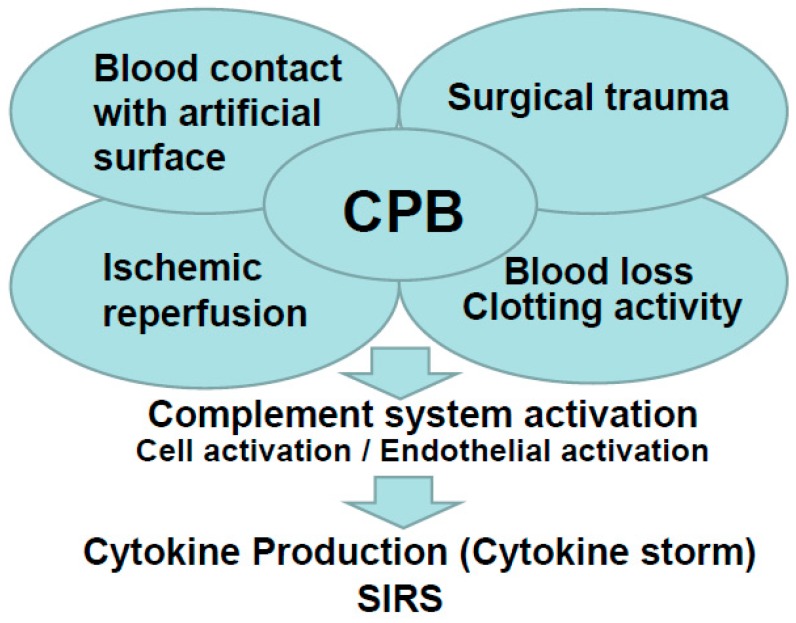
Summary of the inflammatory response in cardiopulmonary bypass (CPB).

Our recent report showed that CPB leads to a cytokine release and major organ damage in a rat CPB model [24,25,26,27,28]. In this review, we will introduce our study focusing on the inflammatory response in CPB. Figure 1 summarizes the inflammatory response in CPB.

## 2. Evaluation of Inflammation Caused by Cardiopulmonary Bypass in a Small Animal Model

### 2.1. Our Rat Cardiopulmonary Bypass Model

Cardiovascular surgery with CPB is often accompanied by a systemic inflammatory response, significantly affecting postoperative mortality and morbidity [4]. Further research is needed to elucidation of the pathological physiology during CPB. On the other hand, difficulties in clinical study and large animal experiments have made its elucidation difficult. In this situation, it is desirable to have a small animal CPB model [24,25,26,27,28], which enables continue experiments, to research the mechanism of vital reaction during CPB.

**Figure 2:**
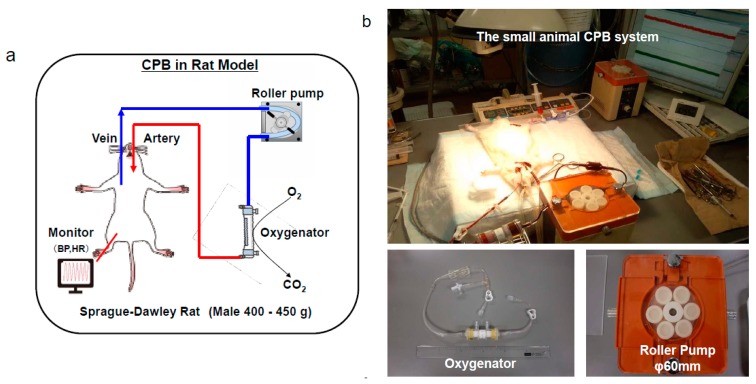
The small animal cardiopulmonary bypass (CPB) model. A polypropylene membranous oxygenator with a membrane area of 0.03 m^2^, a polyvinyl chloride tubing line, and a roller pump are shown [28]. (**a**), schema, (**b**), actual situation.

Male Sprague-Dawley rats (400–450 g), 14–16 weeks old, were used. After the animals were anesthetized with 5.0% isoflurane mixed oxygen enriched air inhalation with a vaporizer, they were placed in the supine position, and a rectal temperature probe was then inserted. Following orotracheal intubation using a 14G catheter (Terumo Corp, Tokyo, Japan), the animals were mechanically ventilated under 40% of oxygen fraction with a Model 687 respirator (Harvard Apparatus Ltd., Edenbridge, Kent, UK) providing volume-controlled ventilation at a frequency of 70/min, with tidal volume of 8–10 mL/kg body weight. Isoflurane 2.0–2.5% was used to maintain anesthesia, and the rectal temperature was kept at 35–36 °C. The right femoral artery was cannulated with SP-31 polyethylene tubing (Natsume Seisakusho Co. Ltd., Tokyo, Japan) for arterial blood pressure monitoring using a Power-Lab system (Model ML870, AD Instruments Japan Inc, Nagoya, Japan). SP-55 polyethylene tubing (Natsume Seisakusho Co. Ltd.) was used to cannulate the left common carotid artery as the arterial return cannula for the CPB system, and heparin sodium (500 IU/kg) was given through this cannula. A 16G cannula (Togo-medkit Co. Ltd., Tokyo, Japan) was passed through the right internal jugular vein advanced into the right atrium as the conduit for venous uptake. The CPB system consisted of a roller pump (MP-3, Tokyo Rikakikai Co., Ltd., Tokyo, Japan) a miniature membrane oxygenator (Senko Medical Instrument Mfg. Co., Ltd., Tokyo, Japan), and polyvinyl chloride tubing line (Senko Medical Instrument Mfg. Co., Ltd.). CPB circuit was primed by 3 mL of sodium bicarbonate, 3 mL of mannitol, 8 mL of Ringer’s solution, and 1 mL (1000 IU) of heparin. Animals in which the hemoglobin level declined to less than 7 g/dl at any point were excluded from the study. Figure 2a,b shows the rat CPB model.

### 2.2. Hyperoxia Promotes the Inflammatory Response during Cardiopulmonary Bypass

In a previous study has shown that, among patients admitted to intensive care units following resuscitation from cardiac arrest, the normoxia control group had significantly lower in-hospital mortality than the hyperoxia (arterial pressure of oxygen (PaO_2_) of 300 mm Hg or greater) control group [29]. A recent study showed that hyper oxygen condition induces oxidative cell damage by promoting the formation of reactive oxygen species (ROS) [30] and the inflammatory cytokines expression [31]. However, PaO_2_ is controlled at high levels during CPB in clinical sites [31]. We hypothesized that hyperoxia aggravates the systemic inflammation and causes organ damage during CPB. We considered that appropriate normoxia control would lead to a reduction of inflammatory cytokine levels, providing protective effects against organ damage during CPB. To test our speculation, the effects of normal and high levels of PaO_2_ on the levels of cytokines (TNF-α, IL-6, and IL-10) and organ damage enzymes (lactate dehydrogenase (LDH), aspartate aminotransferase (AST), and alanine aminotransferase (ALT)) were investigated in a rat CPB model. Moreover, the lung wet-to-dry weight (W/D) ratio was measured as an index of edema. In addition, dihydroethidium (DHE) staining was performed to detect superoxide production in the liver and lung tissues. 

The experimental design is shown below. The animals were randomly divided into three groups: SHAM group received surgical procedure only without CPB, and PaO_2_ was controlled at 100–150 mmHg in the experiment period (*n* = 5), hyperoxia CPB group, PaO_2_ was controlled at greater than 400 mmHg during CPB (*n* = 7), and normoxia CPB group PaO_2_ was controlled at 100–150 mmHg during CPB (*n* = 7). In all experiments, CPB perfusion flow was controlled at 60–70 mL/kg/min. During the experimental period, the partial pressure of arterial carbon dioxide (PaCO2) was ordinarily controlled at 35–45 mmHg in all groups.

Blood samples were collected with every 60 min (pre-CPB, 60 min after initiation of CPB and end-CPB). Plasma levels of cytokines were measured by enzyme-linked immunosorbent assay (Quantikine^®^ ELISA kit, R&D systems, Minneapolis, MN, USA,) and multiplex suspension array (Bio-PlexTM Assay Kits, Hercules, CA, USA). The concentrations of LDH, AST, and ALT were measured by automated colorimetry from plasma samples (DRI-CHEM 7000 Analyzer, FUJIFILM, Kanagawa, Japan). All animals were sacrificed at the end of experiments by potassium chloride injection into the heart, and the left lung was harvested and divided into three parts [25]. The lung block was weighed before and after desiccation for 72 h in a dry oven at 70 °C for the calculation of the W/D ratio [25]. Additionally, a part of the liver and the right lung were placed in cold PBS buffer and then embedded in a dry ice acetone method for cryo-sectioning. The frozen segments were cut into 7-μm-thick transverse sections that were then placed on glass slides [25]. DHE stain solution (Wako Pure Chemical Industries, Ltd., Osaka, Japan) diluted 30,000 times with dimethyl sulfoxide was topically applied to each tissue section. The slides were incubated in a light-protected chamber at 37 °C for 30 min. Images of the tissue sections were obtained using a fluorescence microscope (wavelength 594 nm, exposure time 80 ms) with a rhodamine filter [25]. Fluorescence intensity, which positively correlates with the amount of superoxide generation, was determined in the liver and lung tissues using software (Image J, v1.60, National Institutes of Health, Bethesda, MD).

All value is presented as means ± standard error (SE). Comparisons among groups were performed using analysis of variance (ANOVA). Fisher’s PLSD post hoc test was used for subsequent comparisons between groups at the same time points. Statistical analyses were performed with Stat View 5.0 (Abacus Concepts, Berkeley, CA, USA). Statistical significance was assumed when the *p* value was less than 0.05. The following studies underwent similar statistical processing.

**Figure 3:**
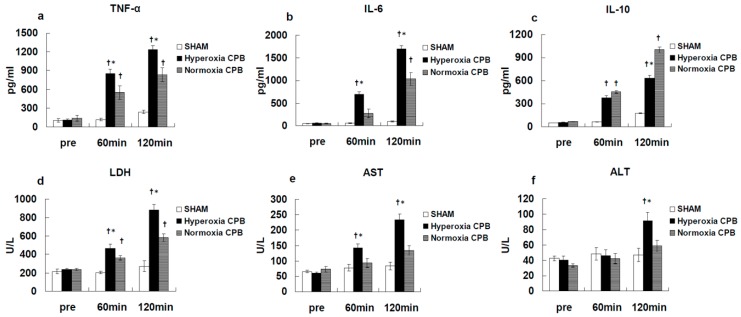
Plasma levels of tumor necrosis factor (TNF)-α (**a**), interleukin (IL)-6 (**b**), IL-10 (**c**), lactate dehydrogenase (LDH) (**d**), aspartate aminotransferase (AST) (**e**), alanine aminotransferase (ALT) (**f**) [25]. † *p* < 0.05 versus the SHAM group, * *p* < 0.05 versus the Normoxia CPB group at the same point in time.

Figure 3 shows the results [25]. Before CPB, the plasma levels of inflammatory and organ damage enzymes were not significantly different among the SHAM, hyperoxia CPB, and normoxia CPB groups. In the SHAM group, plasma inflammatory and organ damage enzymes remained unchanged during the experiment. In the hyperoxia CPB group, pro-inflammatory cytokines (TNF-α and IL-6) increased significantly, reaching a maximum at the end of CPB. However, in the normoxia CPB group, the increases in the pro-inflammatory cytokines were significantly suppressed by approximately 40% compared to the hyperoxia CPB group (Figure 3a,b). On the other hand, in the normoxia CPB group, IL-10 significantly increased, reaching a maximum at the end of CPB, approximately 60% higher in comparison to the hyperoxia CPB group at the end of CPB (Figure 3c).

**Figure 4:**
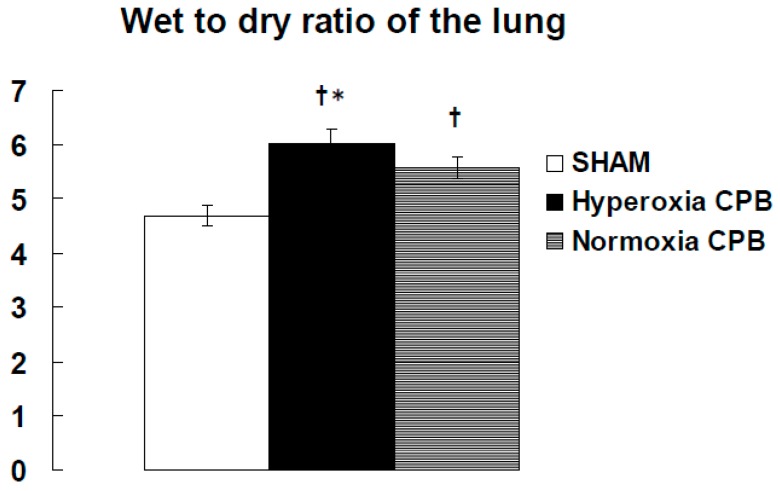
The wet-to-dry weight (W/D) ratio of the lung at the end of experiment [25]. † *p* < 0.05 versus the SHAM group, * *p* < 0.05 versus the Normoxia CPB group.

**Figure 5:**
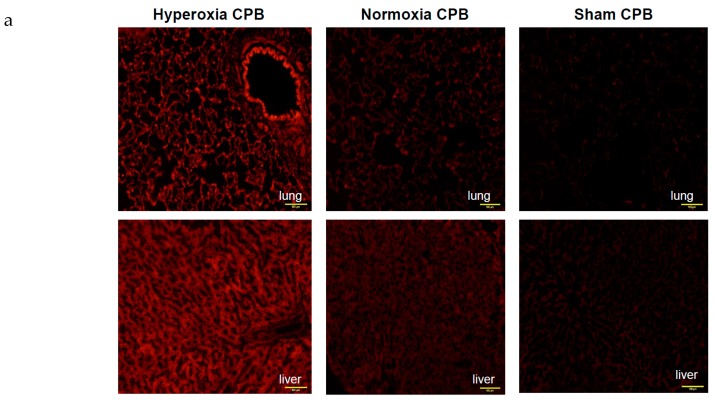

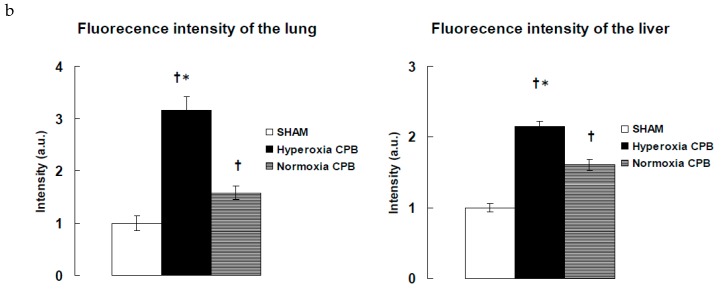
Detection of superoxide production in liver and lung tissues. (**a**) Representative examples of in situ detection of superoxide in each group’s lung and liver. Confocal microscope sections of organ are labeled with the fluorescent oxidative dye dihydroethidium [25]. Scale bar: 100 μm. (**b**) Mean fluorescence intensity after deducting the values of SHAM. † *p* < 0.05 versus SHAM group, * *p* < 0.05 versus Normoxia CPB group [15]. a.u.: arbitrary units.

In the hyperoxia CPB group, the levels of LDH, AST, and ALT increased significantly 60 min after CPB initiation and increased further at the end of CPB. On the other hand, in the normoxia CPB group, the elevated levels of organ damage enzymes were significantly suppressed by approximately 50% at the end of CPB compared to the hyperoxia CPB group (Figure 3d–f). Neither AST nor ALT levels changed significantly during CPB from the pre-CPB levels in the normoxia group. The CPB groups showed significantly higher W/D ratios than the SHAM group (Figure 4). Although, the increase in the W/D ratio was significantly suppressed in the normoxia CPB group compared to the hyperoxia CPB group. DHE staining in the lung and liver tissues was strikingly enhanced in the hyperoxia CPB compared to the normoxia CPB group (Figure 5a,b), this data suggesting greater superoxide production with a hyperoxia condition management during CPB.

### 2.3. Effect of Blood Plasma Substitute Priming on the Systemic Inflammation and Lung Edema Following Cardiopulmonary Bypass

Recently, a new 6% hydroxyethyl starch (HES) with a medium molecular weight (130 kDa) and a very low substitution degree (0.4) was used as a blood plasma substitute (6% HES 130/0.4; VOLUVEN^®^, Fresenius AG, Bad Homburg, Germany). This HES has already been approved in many countries for general fluid replacement. It has also been reported that 6% HES 130/0.4 has pharmacodynamics and pharmacokinetic advantages, such as deceased tissue storage, rapid plasma elimination, and low impact on the blood coagulation system [32,33]. In addition, recent studies have shown that fluid replacement with HES 130/0.4 reduced the inflammation during gastroenterological surgery [34]. We hypothesized that 6% HES 130/0.4 as CPB priming solution would attenuate the systemic inflammatory response with a reduction of pro-inflammatory cytokine levels, providing protective effects against organ tissue damage during CPB [35]. Therefore, in a previous study [16], the effectiveness of HES as CPB priming solution was examined. Plasma levels of TNF-α, IL-6, and colloid osmotic pressure (COP) were investigated in the rat CPB model. Additionally, the lung tissue W/D ratio was studied. 

The animals were randomly divided into three groups: SHAM group (*n* = 5), Ringer’s acetate CPB group (*n* = 7), and HES 130/0.4 CPB group (*n* = 7). The SHAM group received surgical preparation only without CPB. In the Ringer’s acetate CPB group, the CPB circuit was primed with Ringer’s acetate solution (Veen F^®^ Kowa Co., Ltd.), and in the HES 130/0.4 CPB group, the CPB system was primed with 6% HES 130/0.4 (VOLUVEN^®^, Fresenius Kabi Japan K.K.). CPB perfusion flow was controlled at 60–70 mL/kg/min. Respectively, the PaCO_2_ and PaO_2_ controlled at 35–45 and 300–400 mmHg. Blood samples were collected at five defined time points, pre-CPB, 30 min, 60 min, 90 min, and 120 min (end-CPB). The 0.9% NaCl was used in fluid replacement management. The 0.9% NaCl was injected by 0.5 mL at the blood sampling timing (total injection volume of 2.5 mL during the experiment). 

**Figure 6:**
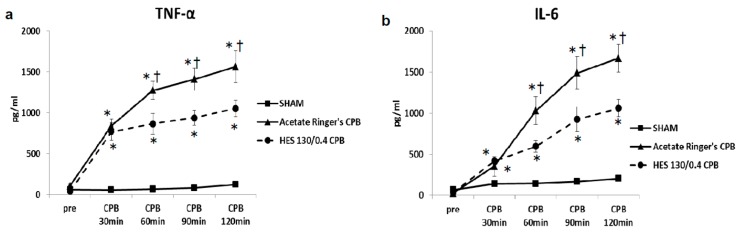
Plasma TNF-α (**a**) and IL-6 (**b**) [35]. * *p* < 0.05 versus the SHAM group, † *p* < 0.05 versus the HES 130/0.4 CPB group at the same point in time.

**Figure 7:**
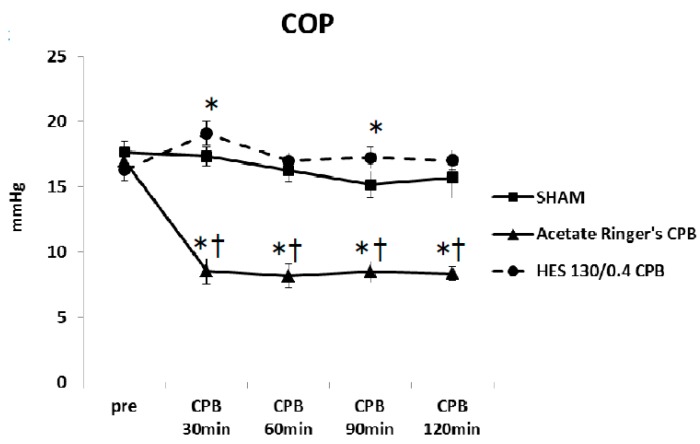
Colloid osmotic pressure (COP) during the experiment [35]. * *p* < 0.05 versus the SHAM group, † *p* < 0.05 versus the HES 130/0.4 CPB group at the same point in time.

**Figure 8:**
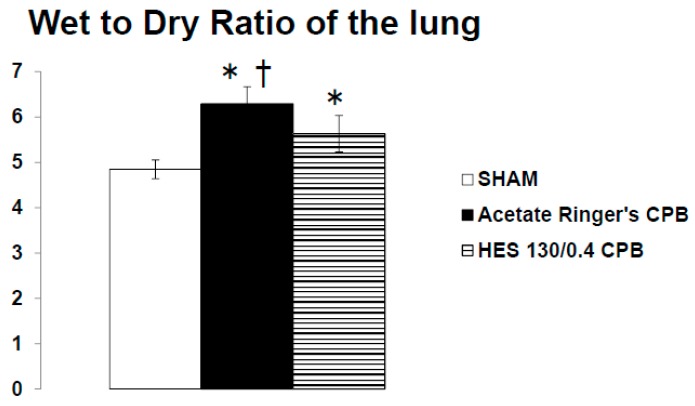
The wet-to-dry ratio of the lung at the end of experiment [35]. * *p* < 0.05 versus the SHAM group, † *p* < 0.05 versus the HES 130/0.4 CPB group.

Pre CPB, the plasma levels of TNF-α and IL-6 were not significantly different among the SHAM, Ringer’s acetate CPB, and HES 130/0.4 CPB groups. Plasma levels of TNF-α and IL-6 remained unchanged during the experimental periods in the SHAM group. In the Ringer’s acetate CPB group, TNF-α and IL-6 increased significantly, reaching a maximum at the end of CPB. However, in the HES 130/0.4 CPB group, the increases in the pro-inflammatory cytokines were significantly suppressed by approximately 35% compared to the Ringer’s acetate CPB group (Figure 6a,b). In addition, it was possible to preserve normal plasma COP levels in the HES CPB group during the experiment (Figure 7). The introduce CPB groups showed significantly higher W/D ratios than the SHAM group (SHAM 4.85 ± 0.08, Ringer’s acetate CPB 6.29 ± 0.14, HES 130/0.4 CPB 5.63 ± 0.15) (Figure 8). On the other hand, the increase in the W/D ratio was significantly suppressed in the HES 130/0.4 CPB group compared to the Ringer’s acetate CPB group.

### 2.4. Method of Suppressing the Inflammatory Response during Extracorporeal Circulation

In a previous article, we examined the protective effect of hydrogen gas (H_2_) in a rat CPB model [24]. We hypothesized that H_2_ insufflation would attenuate the systemic inflammation with a reduction of cytokine levels, providing protective effects against organ damage during CPB. 

The animals were randomly divided into three groups: SHAM group (*n* = 5), CPB group and CPB + H_2_ group (*n* = 7), in which H_2_ was given into an oxygenator during CPB for 60 min. In the CPB + H_2_ group (*n* = 7), H_2_ was added into the membranous oxygenator during CPB at a concentration of 1.4 % (O_2_ flow: H_2_ flow = 1:1). Blood samples were collected pre and 20 and 60 min after the initiation of CPB (end of CPB). Plasma cytokine levels (TNF-α, IL-6, IL-10) were measured. The W/D ratio of the lung was also measured end of experiment. 

**Figure 9:**
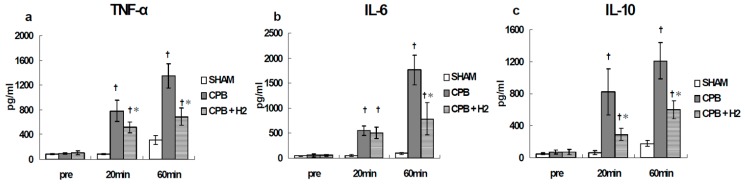
Plasma TNF-α (**a**), IL-6 (**b**), and IL-10 (**c**) [24]. † *p* < 0.05 versus the SHAM group, * *p* < 0.05 versus the CPB group at the same point in time.

In the CPB group, the cytokine levels increased significantly 20 min after CPB initiation and increased further at the end of CPB compared with the SHAM group. However, in the CPB + H_2_ group, such increases were significantly suppressed at the end of CPB (Figure 9).

We suggest that H_2_ is a possible new potential therapy for counteracting CPB-induced systemic inflammation. H_2_ insufflation may attenuate the hyperoxia induced formation of ROS and cytokines through the antioxidant effects. In addition, efforts to control inflammation in the extracorporeal circulation include new methods for directly removing leucocytes and cytokines [36] and development of new coatings and drugs [37]. The strategy of enhancing the biocompatibility of extracorporeal circulation devices and suppressing inflammation will continue in the future.

## 3. Summary

The present study proved that the pro-inflammatory cytokines significantly increased more in the high oxygen partial pressure condition CPB [25]. Furthermore, the lung tissues in the hyperoxia control CPB had higher W/D ratio than the physiologic oxygen partial pressure control CPB, and, therefore, they are presumed to have formation of edema. Additionally, DHE staining for generation of superoxide indicated that there was a striking increase in the liver and lung tissues in the high oxygen partial pressure condition CPB. Several studies have shown that the surfaces of the artificial material activate platelets, leucocytes, and the immune complement system. Activated monocyte-macrophage lineage release cytotoxic agents and ROS associated with the systemic inflammatory response and damage to principal organs [38,39]. There has been a study that showed that hyperoxic condition exposure induces oxidative stress that may activate necroptosis, which is involved in the pathology of hyperoxic acute lung injury [40].

Our hypothesis is that hyperoxia condition causes an increased generation of ROS and confounds the systemic inflammation and damage to principal organs during CPB. The primary product in cellular ROS production is the anion of superoxide. As shown in the present our study, DHE stain fluorescence was used to detect superoxide intracellularly. This is an extensively used method for detection of superoxide [41]. The present data show that the DHE stain fluorescence of the lung and liver tissues was conspicuously enhanced in the oxygen excessive supply condition during CPB.

Additionally, in our study [35], a blood plasma substitute (6% HES 130/0.4) was used as a CPB system priming solution, and the impacts on the systemic inflammatory response and lung edema following CPB were analyzed in a rat model. It was demonstrated that biochemical markers and cytokines for organ damage were effectively suppressed in the HES 130/0.4 CPB group compared to those in the Ringer’s acetate CPB group during CPB in the rat model. The lungs of rats in the Ringer’s acetate CPB group had a higher W/D ratio at the end of experiment than the HES 130/0.4 CPB group and are, therefore, presumed to have formation of edema. Maintenance of a normal COP led to suppression of the systemic inflammatory response and lung edema in the HES 130/0.4 CPB group. The mechanism of the anti-inflammatory effects of 6% HES 130/0.4 in CPB is to maintain COP. Maintenance of a normal COP by HES 130/0.4 suppresses vascular hyperpermeability, further reducing damage to the vascular endothelium and cells of organs. In current clinical research, rehydration therapy with HES 130/0.4 significantly improved tissue oxygenation in patients undergoing gastroenterological surgery [42]. Contrastingly, an equivalent amount of a crystalloid fluids were associated with a striking deterioration of cellular tissue oxygenation. Adverse effects of crystalloid fluids massive infusions were explained by the fact that crystalloid fluids were mainly distributed in the intercellular cement, resulting in reduced interstitial COP and, later, endothelial and cell tissue edema [42]. In addition, there is evidence from animal studies suggesting that COP decrease causes shedding of the endothelial glycocalyx layer so as to significantly increase the capillary leak of albumin and possibly of other plasma proteins [43]. Maintenance of a normal COP suppresses vascular hyperpermeability, further reducing damage to the endothelial glycocalyx layer. Recently, some papers have reported that CPB-induced inflammation is associated with glycocalyx degradation [44]. HES 130/0.4 has the potential to improve organ tissue microcirculation. On the other hand, in the latest review, it was said that there is almost no clinical difference between colloidal solution and crystalloid solution in critically ill patients [45]. The certainty of the evidence may improve with inclusion of ongoing studies in future updates.

From another point of view, it is time to fundamentally review the method of extracorporeal circulation and the surgical procedure for inflammation control in cardiac surgery. It is thought that it is necessary to review the surgical procedure without regard to the conventional method. More recently, minimally invasive cardiac surgery and minimally invasive extracorporeal circulation technologies are actively performed [46,47], and improvement of patient outcomes is expected. Long-duration follow-up data are needed to identify any long-term advantage. In addition, drugs such as steroids are also administered for cardiac surgery, aiming at suppressing inflammation and protecting vital organs [48,49]. There has been considerable research on this subject, but the results are unclear [50,51,52]. Furthermore, the effect of inhibiting inflammation by preoperative plasma-thrombo-leukocyte apheresis has also been reported in an animal experiment [53]. In clinical sites, the focus has been on the potential of leukocyte removal, and anti-inflammatory strategies used to reduce CPB-related complications have been analyzed in a systematic review of randomized, controlled trials [54]. This analysis suggests use of a leukocyte filter [55,56]. Proactive intervention from the preoperative phase may also be necessary for inflammation control. There are other groups that have evaluated inflammation in small animal extracorporeal circulation models, as well as our group [57,58]. Wang and colleagues reported the anti-inflammatory effects of ozone [59].

In the future, it is important to develop high biocompatibility devices, eliminate active oxygen generation, use anti-inflammatory drugs, and remove direct inflammatory substances to suppress inflammation during ECC. Basic research on the biological response to extracorporeal circulation is very important, and there is no doubt that a physiologically close circulation can suppress inflammation. On the other hand, it is generally known that CPB induces myocardial damage. Furthermore, the biological properties of myocardial injury after cardiac arrest is introduced are strongly associated with apoptosis and increased inflammation of myocardial tissue, causing transient cardiac dysfunction. At present, basic research using small animals was mainly conducted during CPB, and no studies evaluated after CPB. Further studies are needed to assess the apoptosis and inflammation in myocardial tissue after CPB.

## 4. Ethics Approval and Consent to Participate

This study was approved by the National Cerebral and Cardiovascular Center Research Institute Animal Care and Use Committee and the Niigata University of Health and Welfare Animal Care and Use Committee (ethical code: 30009,28010). All procedures met the National Institutes of Health guidelines for animal care.

## Data Availability

Data sharing is not applicable to this article as no datasets were generated or analyzed during the current study.

## Abbreviations

ECC	Extra-corporeal circulation
CPB	Cardio-pulmonary bypass
W/D	Wet-to-dry
DHE	Dihydroethidium
ROS	Reactive oxygen species (ROS)
PaO_2_	Arterial pressure of oxygen
PaCO_2_	Arterial pressure of carbon dioxide
SE	Standard error
ANOVA	Analysis of variance
PLSD	Protected least significant difference
COP	Colloid osmotic pressure
